# Clinical Ethics Consultation: Examining how American and Japanese experts analyze an Alzheimer's case

**DOI:** 10.1186/1472-6939-9-2

**Published:** 2008-01-29

**Authors:** Noriko Nagao, Mark P Aulisio, Yoshio Nukaga, Misao Fujita, Shinji Kosugi, Stuart Youngner, Akira Akabayashi

**Affiliations:** 1 Department of Biomedical Ethics, Graduate School of Medicine, Kyoto University, Kyoto, Japan; 2 Center for Biomedical Ethics and Law, Graduate School of Medicine University of Tokyo, Tokyo, Japan; 3 Department of Bioethics, Case Western Reserve University, Ohio, USA; 4 Department of Biomedical Ethics, Graduate School of Medicine University of Tokyo, Tokyo, Japan

## Abstract

**Background:**

Few comparative studies of clinical ethics consultation practices have been reported. The objective of this study was to explore how American and Japanese experts analyze an Alzheimer's case regarding ethics consultation.

**Methods:**

We presented the case to physicians and ethicists from the US and Japan (one expert from each field from both countries; total = 4) and obtained their responses through a questionnaire and in-depth interviews.

**Results:**

Establishing a consensus was a common goal among American and Japanese participants. In attempting to achieve consensus, the most significant similarity between Japanese and American ethics consultants was that they both appeared to adopt an "ethics facilitation" approach. Differences were found in recommendation and assessment between the American and Japanese participants. In selecting a surrogate, the American participants chose to contact the grandson before designating the daughter-in-law as the surrogate decision-maker. Conversely the Japanese experts assumed that the daughter-in-law was the surrogate.

**Conclusion:**

Our findings suggest that consensus building through an "ethics facilitation" approach may be a commonality to the practice of ethics consultation in the US and Japan, while differences emerged in terms of recommendations, surrogate assessment, and assessing treatments. Further research is needed to appreciate differences not only among different nations including, but not limited to, countries in Europe, Asia and the Americas, but also within each country.

## Background

Ethics consultation is a service provided by an individual or a group to help patients, families, surrogates, healthcare providers, or other involved parties address uncertainty or conflict regarding value-laden issues that emerge in healthcare [1]. In the United States (US), ethics consultation services have rapidly expanded since the 1980s; currently, this service is provided at all hospitals with 400 or more beds. Thirty-six thousand cases are requested yearly, involving roughly 29,000 consultants [2]. Several studies on ethics consultation have been published in the US, including discussions based on case studies [3,4], evaluations of ethics consultation [5,6], and analyses of consultation recommendations and their relevant factors [7,8]. Recently reports on ethics consultation have also been published in countries such as Australia, Canada, Italy, Japan, Germany, Norway, Switzerland and the UK [9-15]. These reports highlight a diversity in modality of clinical ethics consultation among and even within different nations. However, there is very little international comparative research that identifies similarities and differences in ethics consultation.

The practice of ethics consultation has often depended on clinical ethical judgments and practical knowledge. Even in the US, 95% of the individuals performing ethics consultation have not completed any formal graduate level training [2]. Comparisons based on case studies are therefore an appropriate means of beginning to assess the similarities and differences in practical knowledge that guide ethics consultation in diverse international contexts.

The objective of this study was to explore how American and Japanese experts analyze an Alzheimer's case regarding ethics consultation. This case focused on the nutritional management of an elderly Alzheimer's patient. We used this case because a review of the literature showed that it has certain key elements that are likely to provoke dilemma or controversy among healthcare practitioners: the patient is incompetent, there are questions about whether or not to opt for terminal care, and disagreements easily arise among the interested parties [16,17]. In this paper, we analyze the recommendations and approaches of ethics consultants from the US and Japan concerning this case and also discuss the legal and institutional aspects of terminal care issues that are presented. Because it is necessary to identify practical knowledge in ethics consultation, this study may assist in educating ethics consultants.

## Methods

### Study Design

We chose experts from both the US and Japan and conducted our research from July to August 2006 using a questionnaire survey, followed by expert interviews. We divided the participants into American and Japanese teams, had them examine the case of an Alzheimer's patient, and conducted follow-up interviews. Participants were told to approach the case as if it occurred in their respective countries. We conducted a content analysis of the teams' approaches and conclusions. All the interviews were performed by three authors of this paper (NN, YN, MF). The study was approved by the Ethics Committee of the Graduate School of Medicine, University of Tokyo.

### Participants

Participants were recruited from among researchers who have published several reports on ethics consultation, medical ethics, and bioethics in academic journals. Many of the individuals practicing ethics consultation in the US are healthcare workers, chaplains, or ethicists [2], while ethics consultation in Japan is often performed by physicians and ethicists. We therefore selected four experts from the fields of medicine and ethics from the US and Japan as participants (Table 1). All participants were male. We explained to all participants their role in this study and received their consent in writing.

**Table 1:** Participant's Demographic Information

Attributes	Physician A	Ethicist B	Physician C	Ethicist D
Nationality	United States	United States	Japan	Japan
Specialization	medicine/psychiatry	ethics	medicine/internal medicine	ethics
Professional degree	M.D.	PhD in philosophy	M.D.	MA in philosophy
Affiliation	university	university/hospital	general hospital	university
Number of available hospital beds	university hospital 500–600 beds	polyclinic hospital 700 beds	general hospital 110 beds	university hospital, mid-size general hospital 200–300 beds
Years of experience	about 20	about 11	about 1	about 4
Total number of cases experienced	more than 500	about 250	about five	about 400

The Japanese participants tended to have fewer years of ethics consultation experience and have handled a smaller number of cases. This was because ethics consultation in Japan has only been initiated in recent years. The Japanese participants selected for this study, however, had been active in research and clinical ethics consultation. Physician C, for example, had undertaken research on topics such as advance directives; and ethicist D had undertaken research on medical ethics education. It was therefore appropriate to regard physician C and ethicist D as experts in ethics consultation for the purposes of this explorative study.

### Data Collection

1. Questionnaire: The questionnaire asked participants about their affiliations and ethics consultation experiences, to describe some of the typical cases they have handled, and included questions related to Alzheimer's patients (patient competency, surrogates, and selection of treatment methods). We created an interview guide based on the responses received in the questionnaire.

2. Expert interview: We conducted a semi-structured expert interview with each participant based on the results of the questionnaire. The expert interview was designed to increase our understanding of the technical and practical knowledge of professionals from that specialty. Accordingly, the interviewee is not merely treated as a case, but as an expert within that particular field [18]. During this interview, we asked the participants how they receive requests for ethics consultation and how they assist and advise their clients. We also asked the participants about the Alzheimer's case, including their recommendation, surrogate evaluation, and treatment assessment.

3. Team case study: We had the four experts divide into two respective teams – one from the US and one from Japan. The two teams individually discussed the case before submitting their recommendation in writing. The teams first exchanged their written recommendation for this case and then met to comment on and discuss each other's recommendation. The Japanese and American team members neither knew each other nor had conversations regarding bioethical issues prior to this study.

4. Follow-up interview: We conducted a semi-structured follow-up interview based on the recommendations of the two teams. We interviewed each participant concerning how they developed their recommendation for the Alzheimer's case as a member of a two-party team.

The questionnaire and case were first developed in Japanese and then translated into English. We confirmed the accuracy of translation by performing a native check and back-translation. The interview was done in the participant's native language and was recorded with their informed consent. We interviewed each participant for three to four hours in total.

### Data Analysis

The entire interviews were transcribed verbatim and analyzed using content analysis [18,19]. Initially one of the authors (NN) coded the data by keyword (eg, futility of nutrient treatment, evaluation of terminal stage Alzheimer's disease) and then further coded those keywords into categories (eg, treatment assessments). The sets of keywords and categories along with the initial data were reviewed by two other coauthors (YN, MF). The three authors discussed any discretion regarding the interpretation of the data and reached a consensus.

### Case

The case presented below is fictional, but is based on an actual case from Japan. The case is considered typical of ethics consultation cases in both the US and Japan, based on the data collected from the interview and questionnaire, and review of the published literature [2,20].

#### Patient Details and Consultation Request

A psychiatrist at N City General Hospital visits an ethics consultant with an ethical dilemma and requests consultation services.

#### Explanation by the Physician (Table 2, Figure 1)

**Figure 1:**
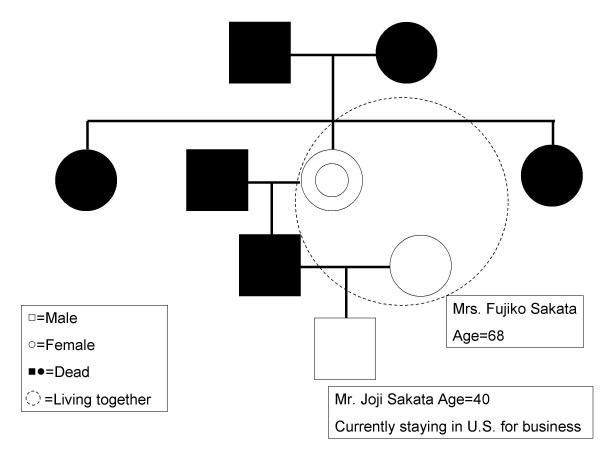
**Family tree of the case**. White square means Male. White circle means Female. Black squares and circles mean Dead. Dot circle signifies 'Living together.'

**Table 2:** Explanation by the Physician

Name and age	Mrs. Mineko Sakata. Age = 92 SEX: Female
Diagnosis	Late-onset Alzheimer's disease.
Chief complaints	disturbance of consciousness, cognitive impairment, and dysphagia
[History and Episode] The patient began to exhibit impairment of memory and orientation in 1998 and has been progressive since. Her family first brought her to our hospital in July 2000 when they discovered she was wandering about aimlessly and screaming in the middle of the night (ambulatory automatism). She was diagnosed with late-onset Alzheimer's disease. The patient fell down at home in September 2005. A local orthopedist found the patient to have a fracture in her left femur. Although the patient did not undergo surgery and was followed closely, she became bedridden, which made it difficult for her family to take care of her at home. The patient's family admitted her to X Elderly Care Facility in October 2005. While the patient was at the X Facility, she remained drowsy throughout the day and night. When addressed in a loud voice, she would open her eyes. She sometimes moans without any comprehensive utterances. The patient could not chew or swallow, which made oral feeding difficult. In July 2006, the patient was transferred to our hospital. Our staff has tried to tube feed her via a nasal gastric tube; but she persistently removes the tube. The patient is currently physically stable and is not considered to be at the end-of-life stage.	
A member of our staff has indicated the possible legal and ethical problems of using only a peripheral intravenous drip (IV), since doing so would lead to a prognosis of death within a few weeks. We therefore did not propose the use of only an IV to her family as a possible therapy. We have also ruled out central venous nutrition (total parenteral nutrition using a central venous catheter), and we did not recommend it in terms of the patient's age, condition and the necessary invasive interventions. As a result, we recommended a gastrostomy (Percutaneous Endoscopic Gastrostomy: PEG) for total enteral nutrition. In the future, we plan to transfer the patient to Y hospital for elderly.	
Mrs. Fujiko Sakata, the patient's daughter-in-law, has expressed that she would not want any other medical treatments if the patient were unable to eat. The patient also has a grandson whose name is Joji, Mrs. Fujiko Sakata's son, and who occasionally comes to visit the patient. His opinion is that a gastrostomy would be allowed if it can prevent his grandmother from dying of starvation. Joji is currently in the US since he has worked there for a long period of time.	

Explanation by the physician is summarized in Table 2 and Figure 1.

#### Family's Explanation

I have taken care of my mother-in-law, Mrs. Mineko Sakata, and I know that her love-of-life derives much from food. I feel that to force her to live without the ability to eat would be inappropriate. For Mineko, the fact that she can no longer eat signifies the end of her life. I really do not want Mineko to have any further invasive medical treatments because she is so old. I am just her daughter-in-law, and the widow of her son, so I'm quite reluctant to make the final decision. Joji, my son, was not very close to his grandmother and did not take much interest in caring for her while she was at home. He also hasn't come to visit her that often here at the hospital. Mineko only has us left as living relatives since her sisters have already passed away.

## Results

The central problem in this case was that there were conflicts of opinion within the family and between the family and the physician. These arose, in part, because the patient had not indicated her own intentions. The results of our analysis showed that because the preference of the patient is unclear in this case, the patient's wishes are discerned by gathering information and establishing a consensus in her best interests. However, the recommendations of the American and Japanese experts differed, based on their surrogate evaluation and treatment assessment. The recommendations of the two teams are as follows:

### 1. Recommendations

#### American team

The patient be provided with "comfort measures only" (do not resuscitate; comfort care) in accord with palliation or comfort as an appropriate goal for care at this time. Members of the care team and Mrs. Fujiko Sakata again attempt to contact Mr. Joji Sakata in the hope that he will come to visit his grandmother in her last days and console his mother.

#### Japanese team

Treatments for oral intake could be explored by a nutrition support team (NST) (if an NST is unavailable, treatment could be explored by a team including an attending nurse and a registered dietitian or speech therapist) combined with the use of a peripheral intravenous drip.

Two differences are clear between these recommendations: contact with the grandson, and the method of treatment. First, the American and Japanese participants differed on whether or not to contact the grandson (daughter-in-law's son). The experts from the US stated that it was important to contact the grandson. The Japanese team, however, assumed that the daughter-in-law was the surrogate and did not advise contacting the grandson. Second, the recommendations of the two teams with respect to the question of appropriate treatment differed. The experts from the US advised ceasing nutrient treatment and recommended palliative care. In contrast, those from Japan advised the provision of nutrient treatment by oral intake with the aid of a NST or a peripheral intravenous drip. To understand these differences, we examined surrogate evaluation and treatment assessment.

### 2. Surrogate Evaluation

All four American and Japanese experts said that the patient of this case was not competent for decision-making. The four experts concurred that it was important to respect the wishes of the surrogate as much as possible provided that he or she was focused on the patient's wishes and not on his/her own values and preferences. For both teams, surrogate evaluation consisted of the common factor of selecting one who could help to identify the patient's wishes.

#### (1) Selection of surrogate

The Japanese team regarded the daughter-in-law, who had a close relationship with the patient, as the key person for surrogate decision-making. However, the American team thought it to be best to identify not only the daughter-in-law but also the grandson, an individual whom they considered best from a "legal standing." The reason behind this is twofold: 1) the grandson is related by blood and thus is legally better fit as a surrogate decision-maker, yet lives far away and has little relationship with the patient, and 2) the daughter-in-law lives close by and has a good relationship with the patient, yet is unrelated by blood and thus has less legal right to make surrogate decisions. In addition, the experts from the US understood that the daughter-in-law wanted to include the grandson in choosing a treatment. Consequently, to select an appropriate surrogate, the American team thought it to be best to contact the grandson. According to physician A:

*I think in the United States, the grandson would have greater legal standing as a surrogate than the daughter-in-law. ... (But on the other hand,) the grandson, from a moral perspective, he really doesn't deserve to have the role of surrogate, so you have this conflict between the law, the literal interpretation of the law and what seems to be right here, and so I would very much try to get the daughter-in-law and the son and the grandson to come to some conclusion together*...

On the other hand, the Japanese team recommended that only the daughter-in-law should be chosen as the surrogate. Given that there is no legal barrier to the daughter-in-law to be a surrogate decision-maker, the experts from Japan were not particularly concerned about contacting the grandson and did not treat this as an important issue. Their reasoning was that the daughter-in-law understood the wishes of the patient and thus can act as a suitable decision-maker. For example, ethicist D considered the daughter-in-law to be the best person given her relationship with the patient.

*Certainly, the daughter-in-law would be the best candidate to make decisions for the patient. I mean they had a good relationship, didn't they. In all and all, the amount of time spent with each other determines a lot about a relationship. It's not like we could just ignore her opinion. If we had to pick between the grandson and her, I would think that she would be the best person*.

### 3. Treatment Assessment

Treatment assessment involved the futility of nutrient treatments and evaluation of terminal disease.

#### (1) Futility of Nutrient Treatment

The experts from the US thought that technical provision of nutrition and hydration was medically ineffective and therefore unnecessary in this case. Physician A stated.

*I think it's futile because preserving life of a severely demented person by putting a surgically implanted tube in them is to me pointless. I would not want this, number one. Number two, there's some medical evidence; there's growing medical evidence that it's not even effective at late stage Alzheimer's (disease) *[21,22].

Based on this supportive evidence, he felt that medically administrated nutrition and hydration simply prolongs the dying process and is meaningless for a patient with an advanced cognitive disease. It should be noted that the two experts from the US were not opposed to assisted oral feeding in the event that the patient became capable of chewing or swallowing.

The two participants from Japan indicated that they considered the technical provision of nutrition and hydration to be psycho-socially beneficial. Ethicist D indicated that he was skeptical of its futility. His opinion was that "physicians in Japan do not always deny medically assisted nutrition and hydration at the terminal stage;" rather, in some cases it is possible to utilize it to satisfy the family's needs. Physician C suggested the following:

*Even if the patient has little awareness, or has a high degree of mental deterioration, or is even bedridden, or does not even speak, I think that it is socially important that the patient be kept alive as long as the patient has family or grandchildren who wish for such*.

#### (2) Evaluation of Terminal-Stage Alzheimer's Disease

The American and Japanese participants varied in their opinions on the futility of nutrient treatment concerning this case. This most likely arose from their different perceptions on the implications of terminal Alzheimer's disease. On the one hand, the Japanese participants believed that a patient's life can be prolonged with a gastrostomy and thus should not be considered to be "end-of-life." On the other hand, the two experts from the US thought that this case applied to end-of-life care based on recent evidence that nutrient treatment and transfusions are not beneficial to prolonging life [21,22]. Thus the two teams disagreed on their interpretation of whether or not this case should be considered to be "end-of-life" and likewise on their recommendations regarding nutrient treatment. The two experts from the US regarded nutrient treatment of terminal-stage Alzheimer's patients with no hope of regaining consciousness as unbeneficial. They regarded terminal Alzheimer's disease, a higher-function brain disorder, as not significantly different from terminal cancer or any other terminal illness. Physician A noted,

I mean people get worse and worse and their prognosis gets worse and worse and that's true with cancer and that's true with Alzheimer's. So I guess what I would say is if an Alzheimer patient has two months to live, why would you want to treat them differently than a cancer patient who has two months to live?

Contrary to this, the Japanese participants regarded nutrient treatment of terminal-stage Alzheimer's patients with no hope of regaining consciousness as beneficial. This was based on the reasoning that terminal-stage Alzheimer's disease is medically different from terminal cancer. Although it is difficult to restore the consciousness of terminal-stage Alzheimer's patients, the maintenance of their somatic functions is within the scope of medical treatment. In the case of terminal cancer, however, there is little hope of restoring physical function even given technical provision of nutrition and hydration. Subsequently, palliative care is reserved primarily for terminal cancer patients in Japan. The two ailments were therefore considered to be significantly different. According to physician C,

*In a case such as this (terminal stage Alzheimer's), the patient may live for two or three more years if nutrients are actively administered using either a PEG (percutaneous endoscopic gatrostomy) or IVH (intravenous hyperalimentation). That being said, no matter how much nutrition you provide to patients with terminal cancer, they can't escape death*....

### 4. Consensus Building

Common characteristics were evident in the two teams' approaches to consensus building among individuals involved in the case such as the patient, healthcare providers and possible surrogate decision-makers. The underlying theme of both teams was to seek out the patient's preferences. Ethicist D stated after a team discussion that the approaches to consensus building were "closer than expected" between the American and Japanese participants.

Interestingly enough, differences arose in approach between physicians and ethicists at first, but then were resolved within each team. That is, the physicians tended to formulate their recommendation from the provided information; the American physician chose palliative care, while the Japanese physician chose peripheral intravenous drip. Conversely, the ethicists had difficulty deciding on a treatment because of a lack of information. When the physician and ethicist of each team discussed the case and decided on their recommendation, the results reflected both of their opinions. For instance, physician C recommended peripheral intravenous drip and ethicist D suggested NST oral intake, yet both were included in the final recommendation.

## Discussion

Establishing a consensus was a common goal among American and Japanese participants. In attempting to achieve consensus, the most significant similarity between Japanese and American ethics consultation teams was that they both appeared to adopt an "ethics facilitation" approach. As discussed in the major American report on ethics consultation, *Core Competencies for Health Care Ethics Consultation*, there are three broad approaches to ethics consultation in the literature, "authoritarian," "pure facilitation," and "ethics facilitation"[23,24] [see Additional file 1].

Differences were found in recommendation and assessment between the American and Japanese participants. In selecting a surrogate, the American participants chose to contact the grandson before designating the daughter-in-law as the decision-maker. Conversely the experts from Japan assumed that the daughter-in-law was the surrogate. It is interesting to note that both teams referred to Joji Sakata differently. The experts from the US usually called him the "grandson," because they tried to understand the case from the perspective of the patient. In contrast, the Japanese team tended to call him the "son" because they perceived the daughter-in-law as the key figure. Another difference was found in the assessment of treatments. In short, the American participants regarded the provision of nutrition and hydration as unbeneficial, based on medical evidence [21,22], while the Japanese participants thought it to be beneficial. Yet another difference was found in each team's take on whether this case should be considered to be "end-of-life" or not.

These differences can be discussed from legal aspects of terminal care. Following the Quinlan's case of 1975, and based on a variety of legal precedents, withdrawing or withholding healthcare intervention from terminal-stage patients is permissible in the US. According to ethicist B, the legal framework concerning end-of-life care in the US can be summarized by the following three points: 1) a patient's right to refuse treatment is firm, 2) a patient who is unable to exercise her right, such as this patient, has that right extended ideally through advance directives and a designated surrogate, and 3) state legislation governs the role of surrogates and/or legal ordering of surrogate decision makers, and such legislation varies from state to state. Under these legal conditions, the important role of ethics consultation in terminal care is first to clarify the wishes or goals of the patient. If the patient's preference has not been stated, the patient is not expected to regain consciousness, and there is no way to ascertain the once competent, now incompetent, patient's wishes, the results of ethics consultation should reflect the best interest of the patient.

In Japan, however, legal standards related to terminal care are currently unclear. There are no Supreme Court precedents concerned with terminal treatment, with the only representative cases having been decided by regional courts. We can summarize the contemporary legal interpretations of terminal care, as based on the work of medical law specialists [25], in the following two points: 1) past decisions merely state the conditions for terminating treatment, and 2) if those conditions are not satisfied, then standard treatments must be provided. This Alzheimer's case does not coincide with the Japanese legal conditions for terminating medical care. Therefore, it is difficult to actively terminate therapy, even nutrient treatment, which only prolongs the life of late-stage Alzheimer's patients in Japan. From a legal standpoint such as this, the Japanese participants tended towards emphasizing the beneficence of the physician for performing the minimal standard treatment, giving greater consideration to the psycho-social aspects of nutrient treatment in this case.

The different evaluations of terminal Alzheimer's disease may also be triggered by the fact that different palliative care systems exist in each country. According to the World Health Organization (2002) [26], "palliative care is an approach that improves the quality of life of patients and their families facing the problems associated with life-threatening illness." This statement presumes that all diseases are targets of palliative care. In the US, hospices are likely to treat most end-of-life diseases, whether they are a type of cancer or not, with palliative care [27]. Criteria have been set up in Japan to establish palliative care wards, but these are limited to particular diseases covered by insurance benefits such as cancer and acquired immunodeficiency syndrome (AIDS); therefore, it is difficult to provide palliative care for many other diseases. As a result, terminal Alzheimer's patients in Japan are often not treated as potential recipients of palliative care but usually receive treatment in mid-size city hospitals, unlike terminal cancer patients. This situation may account for the differences in treatment evaluation between the American and Japanese participants.

This study has several limitations. First, this study was to provide preliminary data on how four experts from the US and Japan approach clinical ethics consultation with a terminal-stage Alzheimer's case. In so it was not a comparison of each country's ethics consultation practices. Second, this study's sample was limited to four experts; others experts from each country could provide a different recommendation and position. Third, the background of the participants was limited to physicians and ethicists; likewise results may differ with consultants with different backgrounds. Fourth, this study used a single case concerning a terminal Alzheimer's patient and results may differ with other cases.

## Conclusion

This descriptive study is the first report to analyze the characteristics of ethics consultation between two American and two Japanese experts. Our findings suggest that consensus building through an "ethics facilitation" approach may be a commonality to the practice of ethics consultation in the US and Japan, while differences emerged in terms of recommendations, surrogate assessment, and assessing treatments. Further research is needed to appreciate differences not only among different nations including, but not limited to, countries in Europe, Asia and the Americas, but also within each country.

## Competing interests

The author(s) declare that they have no competing interests.

## Authors' contributions

NN conceived and designed the study, and collected the data. MA, YN, MF conducted data collection and contributed to the writing of the paper. SK assisted with the data analysis. SY and AA supervised data collection and analysis. All authors read and approved the final manuscript.

## Pre-publication history

The pre-publication history for this paper can be accessed here:



## Supplementary Material

Additional file 1 Three types of approaches of ethics consultation. This explains three types of approaches of ethics consultation. Click here for file

## References

[B1] Aulisio MP, Post SG (2003). Ethics committees and ethics consultation. Encyclopedia of Bioethics.

[B2] Fox E, Myers S, Pearlman RA (2007). Ethics consultation in U.S. hospitals: A national survey. Am J Bioeth.

[B3] Lask B (2003). Patient-clinician conflict: causes and compromises. J Cyst Fibros.

[B4] Rosenbaum JR, Bradley EH, Holmboe ES, Farrell MH, Krumholz HM (2004). Sources of ethical conflict in medical housestaff training: a qualitative study. Am J Med.

[B5] McClung JA, Kamer RS, DeLuca M, Barber HJ (1996). Evaluation of a medical ethics consultation service: Opinions of patients and health care providers. Am J Med.

[B6] Studdert DM, Burns JP, Mello MM, Puopolo AL, Truog RD, Brennan TA (2003). Nature of conflict in the care of pediatric intensive care patients with prolonged stay. Pediatrics.

[B7] Brennan TA (1988). Ethics committees and decisions to limit care. The experience at the Massachusetts General Hospital. JAMA.

[B8] Fox E, Stocking C (1993). Ethics consultants' recommendations for life-prolonging treatment of patients in a persistent vegetative state. JAMA.

[B9] Gill AW, Saul P, McPhee J, Kerridge I (2004). Acute clinical ethics consultation: The practicalities. Med J Aust.

[B10] Coughlin MD, Watts J (1993). A descriptive study of healthcare ethics consultants in Canada: Results of a national survey. HEC Forum.

[B11] Hurst SA (2007). Physician's access to ethics support services in four European countries. Health Care Anal.

[B12] Nagao N, Takimoto Y, Akabayashi A (2005). A survey on the current state of hospital ethics consultation in Japan. Journal of Japan Association for Bioethics.

[B13] Reiter-Theil S (2001). Prat II: Clinical ethics consultation in development. Ethics consultation in Germany: The present situation. HEC Forum.

[B14] Slowther A, Bunch C, Woolnough B, Hope T (2001). Clinical ethics support services in the UK: An investigation of the current provision of ethics support to health professionals in the UK. J Med Ethics.

[B15] Slowther A, Underwood M (1998). Is there a need for a clinical ethics support service in the UK?. J Med Ethics.

[B16] Connelly JE, DalleMura S (1988). Ethical problems in the medical office. JAMA.

[B17] Lo B, Schroeder SA (1981). Frequency of ethical dilemmas in a medical inpatient service. Arch Intern Med.

[B18] Flick U (2006). An introduction to qualitative research.

[B19] Lofland J, Lofland LH (1995). Analyzing social settings: A guide to qualitative observation and analysis.

[B20] McGee G, Caplan AL, Spanogle JP, Asch DA (2001). A national study of ethics committees. Am J Bioeth.

[B21] Finucane TE, Christmas C, Travis K (1999). Tube feeding in patients with advanced dementia. JAMA.

[B22] Gillick MR (2000). Rethinking the role of tube feeding in patients with advanced dementia. N Engl J Med.

[B23] Aulisio MP, Arnold M, Youngner SJ (2000). Health care ethics consultation: Nature, goals, and competencies. A position paper from the Society for Health and Human Values-Society for Bioethics Consultation Task Force on Standards for Bioethics Consultation. Ann Intern Med.

[B24] Society for Health and Human Values-Society for Bioethics Consultation (1998). Core Competencies for Health Care Ethics Consultation: The Report of the American Society for Bioethics and Humanities.

[B25] Mizuno T, Maeda S, Akabayashi A (2005). Shumatuki iryou. Introduction to Biomedical Ethics 1.

[B26] (2002). WHO, "Definition of palliative care,". http://www.who.int/cancer/palliative/definition/en/.

[B27] Stevens LM, Lynm C, Glass RM (2005). Palliative care. JAMA.

