# Supplementary material for: Clinical Ethics Consultation: Examining how American and Japanese experts analyze an Alzheimer's case
Source: BMC Med Ethics. 2008 Jan 29;9:2. doi: 10.1186/1472-6939-9-2 (PMC2268696; doi:10.1186/1472-6939-9-2)
Supplement: Additional file 1 — Three types of approaches of ethics consultation. This explains three types of approaches of ethics consultation. [file 1472-6939-9-2-S1.doc]

**Additional file 1.**

**Three types of approaches of ethics consultation****

**Conclusion 1: Our Societal Context Makes “Ethics Facilitation” an Appropriate Approach to Ethics Consultation**

Two salient features of the U.S. societal context shape an appropriate approach to ethics consultation (18 –22). First, U.S. society is pluralistic, comprising persons of diverse racial, ethnic, religious, and nonreligious backgrounds who hold diverse values. This diversity is mirrored in the clinical setting and is further complicated there by the presence of various professional, institutional, and systemic values. Second, individual persons and communities in our society have the right to pursue different conceptions of the “good life” and to live by their own values. This right does not disappear merely because one becomes a health professional or because one falls ill and becomes a patient. An appropriate approach to ethics consultation must be sensitive to the pluralistic health care setting in which consultation is provided and consistent with the societal value of autonomy.

Societal values are often reflected in law and institutional policy, which are also part of the context that frames ethical issues in contemporary health care settings. Therefore, law and institutional policy also inform a proper understanding of ethics consultation and are widely discussed in the bioethics literature. Many states, for example, have legislation that governs the application of advance directives and outlines procedures for surrogate decision making in the absence of such directives. Similarly, institutions have policies that are applicable in certain types of cases, such as guidelines on life-sustaining treatment or requests for organ or tissue donation. Helping to identify the implications of law and institutional policy for particular cases is another role of ethics consultation in contemporary health care settings.

The bioethics literature covers several possible approaches to ethics consultation (23–25). Most approaches fall between one extreme that might be termed the *authoritarian approach* and another that might be termed the *pure facilitation approach*. For illustrative purposes, we will briefly characterize these two extremes; point out their inadequacies; and outline an alternate approach, *ethics facilitation,* that we believe is appropriate for ethics consultation in our society.

**The Authoritarian Approach**

The defining characteristic of the authoritarian approach to ethics consultation is its emphasis on consultants as the primary moral decision makers. Ethics consultation can be authoritarian with respect to *outcome* or *process*.

Consider the case of a competent, well-informed, adult patient who refuses treatment on religious grounds. Imagine that the ethics consultants are sensitive to the process of consultation and talk to all involved parties, addressing the factual, conceptual, and normative issues raised by the case. The consultants then recommend that the patient be given treatment against his wishes, despite the fact that the patient is competent and well informed, because their substantive values differ from those of the patient. The case, of course, begs for more detail, but it suffices to illustrate an authoritarian approach to the *outcome* of consultation; ethics consultants, as the primary moral decision makers, displace the appropriate moral decision maker, in this case the patient. By misplacing moral decision–making authority, this approach fails to recognize the appropriate boundaries of ethics consultation, as fundamentally established by the rights of individuals in U.S. society.

To illustrate the inadequacies of an authoritarian approach to the process of consultation, consider a case in which a family and health care team disagree over continued treatment of a critically ill adolescent. Suppose the health care team believes that continued treatment is futile, whereas the family hopes for a miraculous recovery. After speaking only to the attending physician, the ethics consultant sides with the health care team and recommends that treatment be discontinued. The consultant reaches his decision on the basis of his interpretation of the controversial concept of “futility” as discussed in the bioethics literature (15, 16). This approach is authoritarian in its *process* because it excludes relevant parties from moral decision making. It fails to open lines of communication between the family and the health care team in order to work toward a consensus that falls within the boundaries set by societal values, law, and institutional policy.

**The Pure Facilitation Approach**

The sole goal of the pure facilitation approach is to forge consensus among involved parties. Imagine that consultants facilitate consensus between a patient’s family and the health care team to override the wishes of the patient as expressed in a valid advance directive. The patient has become unconscious, and no other relevant new information has become available. Although the consultants are inclusive and achieve consensus, they do so without clarifying the implications of societal, legal, and institutional values for the case. By placing too much emphasis on facilitating consensus, consultants risk forging a consensus that falls outside acceptable boundaries. In this case, the consensus violates the patient’s right to self-determination.

**The Ethics Facilitation Approach**

We believe that an ethics facilitation approach is most appropriate for health care ethics consultation in contemporary society. The ethics facilitation approach is informed by the context in which ethics consultation is provided and involves two core features: identifying and analyzing the nature of the value uncertainty and facilitating the building of consensus. By “consensus,” we mean agreement by all involved parties, whether that agreement concerns the substantively morally optimal solution or, more typically, who should be allowed to make the decision.

To identify and analyze the nature of the value uncertainty or conflict underlying the consultation, ethics consultants must do the following:

1. Gather relevant data (for example, through discussions with involved parties and examination of medical records or other relevant documents).

2. Clarify relevant concepts (such as confidentiality, privacy, informed consent, and best interest).

3. Clarify related normative issues (such as the implications of societal values, law, ethics, and institutional policy for the case).

4. Help to identify a range of morally acceptable options within the context.

Health care ethics consultants should also help to address the value uncertainty or conflict by facilitating consensus among involved parties (which may include patients, families, surrogates, and health care providers). To do so, they must ensure that the voices of all involved parties are heard, assist the involved individuals in clarifying their own values, and facilitate the building of morally acceptable shared commitments or understandings within the context.

The ethics facilitation approach recognizes the societal boundaries for morally acceptable solutions. In contrast to the authoritarian approach, ethics facilitation emphasizes an inclusive consensus-building process. It respects individuals’ rights to live by their values by not misplacing moral decision–making authority or representing the personal moral views of the consultant only. In contrast to the pure facilitation approach, ethics facilitation recognizes that societal values, law, and institutional policy have implications for a morally acceptable consensus. The ethics facilitation approach is consistent with both the pluralistic context in which ethics consultation is done and the rights of individuals to live by their values, recognizing that there are definite boundaries within which decisions must be made and helping to ensure that these boundaries are not transgressed.

**Aulisio MP, Arnold M, and Youngner SJ. Health care ethics consultation: Nature, goals, and competencies. A position paper from the Society for Health and Human Values-Society for Bioethics Consultation Task Force on Standards for Bioethics Consultation. Ann Intern Med 2000; 133(1): pp 59-62.
